# Molecular Interaction Network Approach (MINA) identifies association of novel candidate disease genes

**DOI:** 10.1016/j.mex.2019.05.031

**Published:** 2019-05-31

**Authors:** Sam Kara, Alaa Hanna, Gerardo A. Pirela-Morillo, Conrad T. Gilliam, George D. Wilson

**Affiliations:** a University of Chicago, Departments of Human Genetics, 920 East 58th St., Chicago, IL, 60637, USA; b Radiation Oncology Department, 3811 W Thirteen Mile Road, Royal Oak, MI, 48073, USA; c Erb Family Molecular & Genetics Laboratory Beaumont Health, 3811 W Thirteen Mile Road, Royal Oak, MI, 48073, USA; d La Universidad del Zulia, Computer Science Department, Laboratories for Computational Models & Languages, and Bioinformatics, Edif. Grano de Oro, Planta Baja, Departamento de Computación, Ave. Universidad con Ave. 22, Maracaibo, 4002, Venezuela

**Keywords:** MINA, Molecular Interaction Network Approach, Autoimmune diseases, Crohn’s disease (CD), Systemic lupus erythematosus (SLE), Rheumatoid arthritis (RA), Psoriasis (PSO), Type-1 diabetes (T1D), Type-2 diabetes (T2D), Celiac disease (CeD), Multiple sclerosis (MS), Association, Molecular network

## Abstract

Molecular Interaction Network Approach (MINA) was used to elucidate candidate disease genes. The approach was implemented to identify novel gene association with commonly known autoimmune diseases [1]. In MINA, we evaluated the hypothesis that “network proximity” within a whole genome molecular interaction network can be used to inform the search for multigene inheritance. There are now numerous examples of gene discoveries based upon network proximity between novel and previously identified disease genes (Yin et al., 2017 [2], Wang et al., 2011 [3], and Barrenas et al., 2009 [4]). This study extends the application of interaction networks to the interrogation of Genome Wide Association studies: first, by showing that a group of nine autoimmune diseases (AuD) genes “seed genes”, are connected in a highly non-random manner within a whole genome network; and second, by showing that the minimal number of connecting genes required to connect a maximal number of AuD candidate genes are highly enriched as candidate genes for AuD predisposing mutations. The findings imply that a threshold number of candidate genes for any heritable disorder can be used to “seed” a molecular interaction network that

• Serves to validate the disease status of closely associated seed genes
• Identifies genes that are highly enriched as novel candidate disease genes
• Provides a strategy for elucidation of epistatic gene x gene interactions

The method could provide a critical toll for understanding the genetic architecture of common traits and disorders.

**Specifications Table**

**Table:** 

Subject Area:	*Biochemistry, Genetics and Molecular Biology* *Immunology and Microbiology*
More specific subject area:	*Describe narrower subject area*
Method name:	*MINA; Molecular Interaction Network Approach*
Name and reference of original method:	[1]
Resource availability:	The Ingenuity Pathway Analysis (IPA) software: http://www.ingenuity.com Pathway Studio 9 MammalPlus (Elsevier B.V.): https://www.pathwaystudio.com/

## Method details

**Fig. 1:**
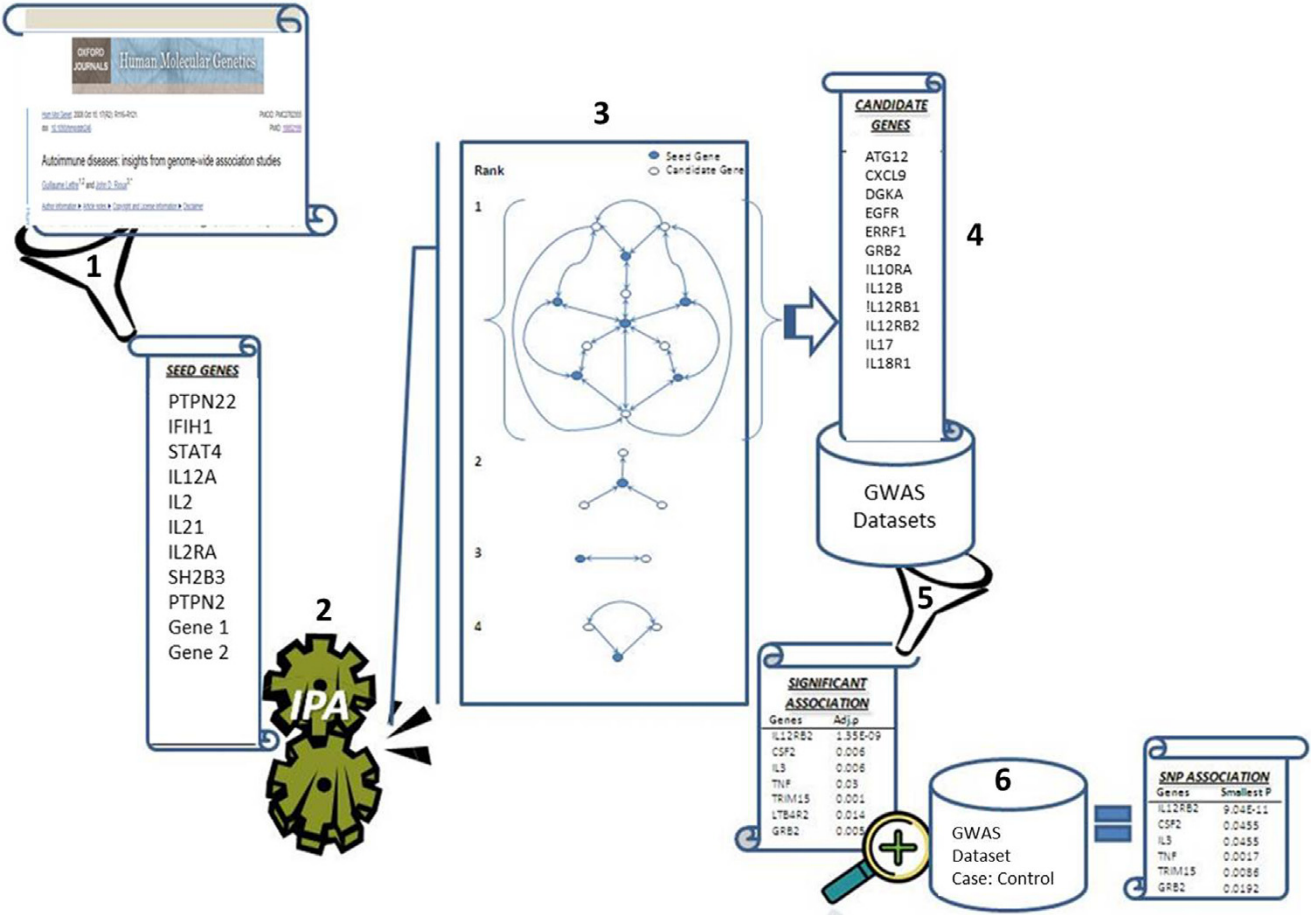
Schematic representation of MINA Workflow. Numbers in bold represent the MINA steps; **1**- Seed genes selected, **2**- Ingenuity Pathway Analysis (IPA) core tool created and score-ranked networks, **3**- Top ranking network selected with the highest p-value, **4**- Candidate genes are identified, **5**- Candidate genes Validation, in primary database, and **6**- Candidate genes replication in different GWAS and/ or new case: control study.

MINA workflow steps are schematically presented in Fig. 1.

1 Seed genes selected from literature.
2 Ingenuity Pathway Analysis (IPA) core tool created and score-ranked networks interconnecting seed genes
3 Largest, highest-scoring network from IPA output selected
4 Candidate Genes (connecting genes), their location, and all their genotyped SNPs are identified.
5 Validation: in primary GWAS dataset
6 Replication: in secondary GWAS dataset and/ or new case: control study.

### MINA study design and candidate seed genes

In MINA, we use “network proximity” to identify a small number of candidate genes that we then “re-evaluated” in the published GWAS studies [1]. Recently, similar approaches using network proximity have been reported [[2], [3], [4]]. Our study design is based on the identification and association analysis of a very small number of candidate genes (relative to a whole genome scan) where the statistical cost of multiple testing is greatly reduced and which allows for cheap and rapid testing candidate genes by testing targeted single nucleotide polymorphisms (SNPs) in case: control study. By lowering the number of SNPs tested we sought to detect candidate AuD genes that were indistinguishable from background noise in the genome wide studies. Genetic studies implicates set of genes that are well established for multipile and overlapping AuD including T1D [[5], [6], [7], [8], [9]]. PSO [10], ankylosing spondylitis [11], and other common heritable disorders [[12], [13]]. A meta-analysis of 18 AuD-GWAS identified a total of nine genes that are common among two or more of the following seven AuD: CeD, CD, MS, RA, SLE, PSO and T1D [5]. These nine identified genes were selected as the “seed genes”.

### Gene and SNPs identification

A gene location was defined to include 100-kilobase up-stream and down-stream of NCBI’s start- and end- gene location. All examined candidate genes SNPs were downloaded from each GWAS database and where assigned to the prospective gene(s) to enable SNP-gene comparisons across multiple databases.

### Graphical representation of gene networks

**Fig. 2:**
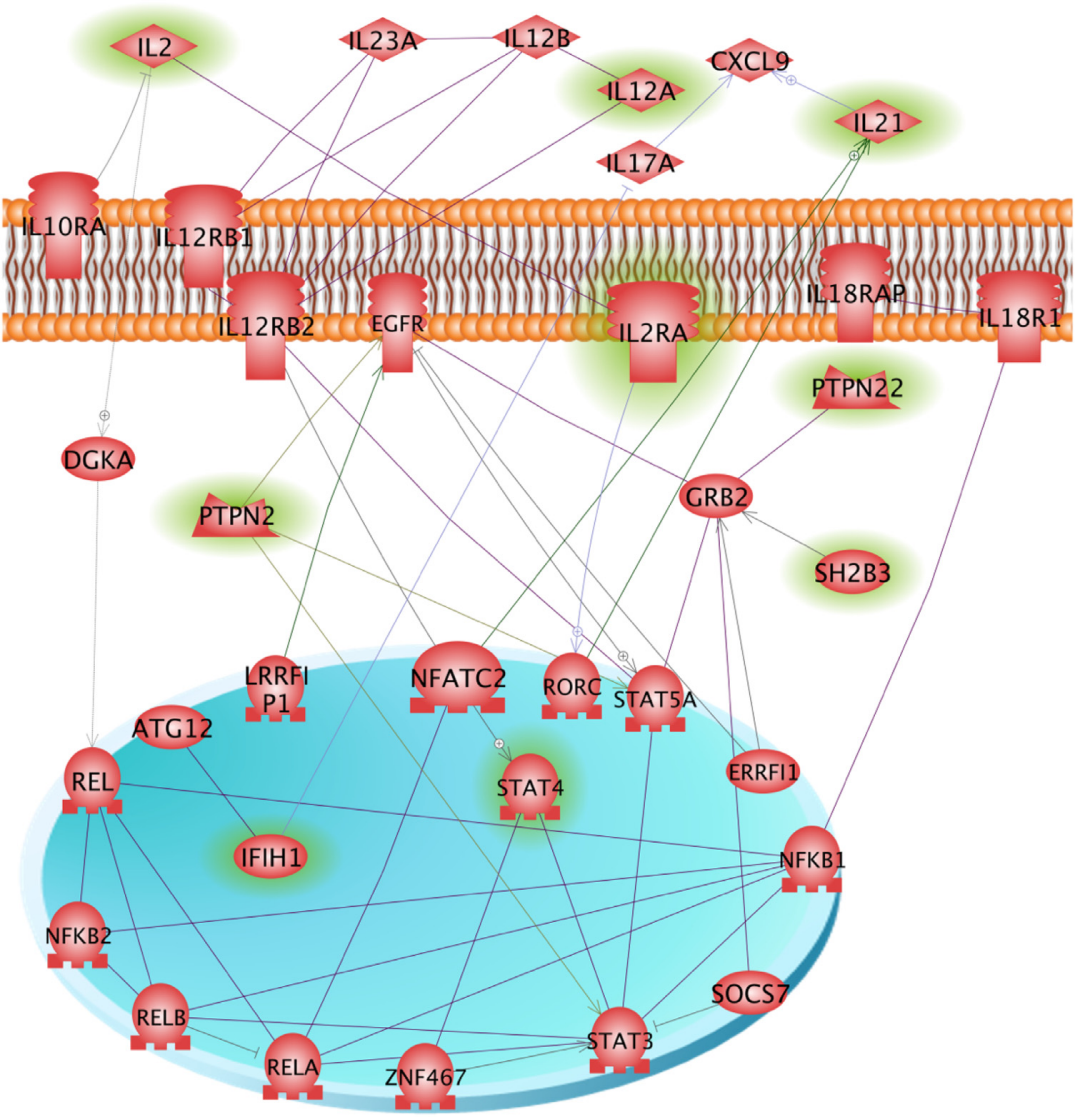
Autoimmune disease specific molecular interaction network. Seed genes (highlighted in green) and candidate genes are displayed in their identified cellular compartment for seven autoimmune diseases (PSO, CeD, CD, MS, RA, SLE and T1D). Genes or gene products are represented as nodes/shapes, and the biological relationship between two nodes is represented as an edge (line). Genes highlighted in green represent the seed genes. All nodes and edges are supported by at least 1 reference from the literature, from a textbook, or from a database that was incorporated into Ingenuity knowledge base. Nodes are displayed using various shapes that represent the functional class of the gene product or molecule class. (For interpretation of the references to color in this figure legend, the reader is referred to the Web version of this article.)

**Fig. 3:**
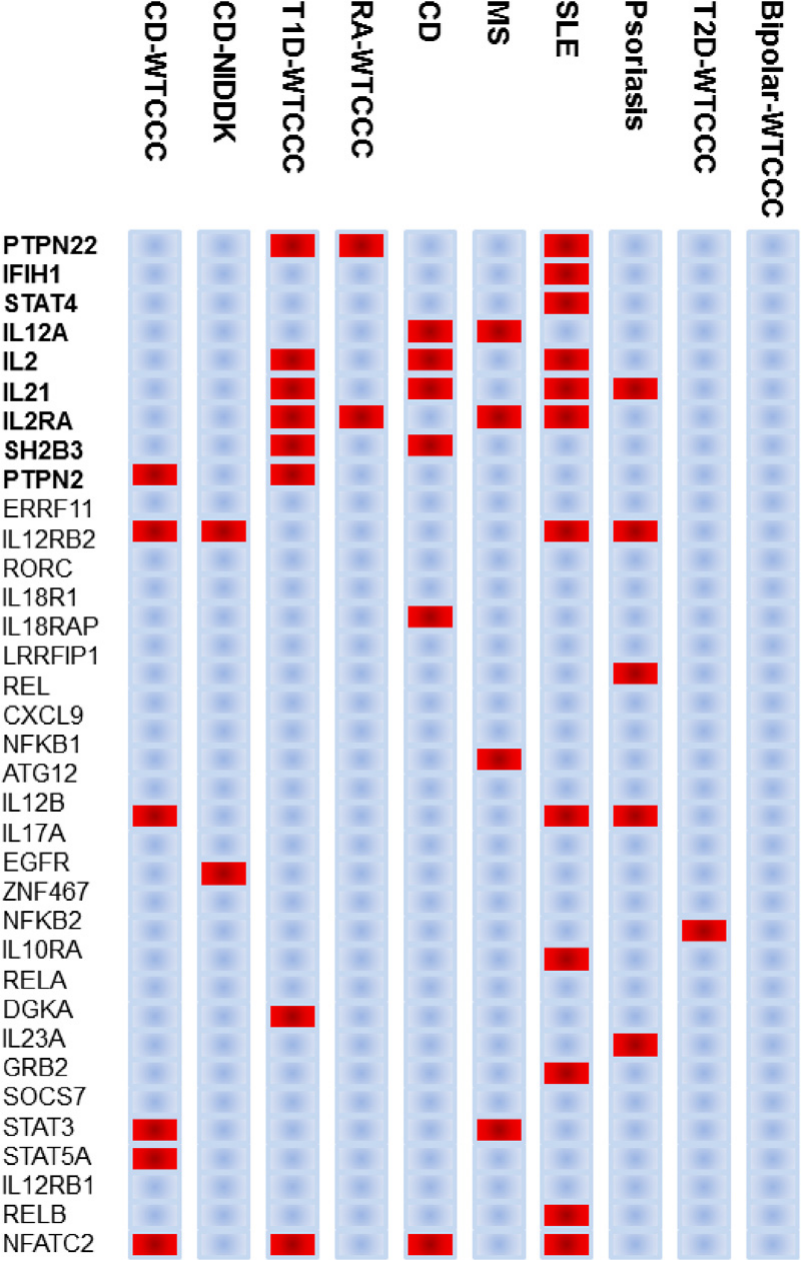
The distribution of the most significant SNPs associated with each disease. Bold genes represent the nine seed genes.

The term ‘network’ is used to refer to a graphical representation of the molecular relationships between genes or gene products. Genes or gene products are represented as nodes (shapes) and the biological relationship between two nodes is represented as an edge (line). In order to facilitate visualization of the seed and network connecting genes we only show the molecular interactions (edges) connecting network members. We tasked the Ingenuity Pathway Analysis (IPA) software to “link together the maximum number of seed genes with a minimal number of connecting genes within the constraints of the default 35-node network”. It is expected that optimization of this problem will include one or more nodes with the network property of a “hub”; i.e., the gene may be selected based on its connections to a large number of molecules rather than biological similarity to the other network genes. Direct interactions refer to actions like “binding”, “cleave”, or “phosphorylate” whereas indirect interactions refer to actions like “activate”, “inhibit”, or “stimulate”. In this study, we only consider direct interaction. We used Pathway Studio 9 MammalPlus (Elsevier B.V.) program for network graphical representation and to reduce the number of edges for clarity of the graphical representation. Fig. 2 represent the AuD specific network and Fig. 3 summarizes the significant association identified for each AuD and the AuD network genes.

### Molecular network building and analysis

We used the IPA software to predict molecular interaction relationships among the nine AuD seed genes and to predict the connecting genes. Seed genes were selected as described above and were uploaded to the IPA program for analysis. The IPA first searches for evidence of direct interaction between seed genes until the maximum number of seed genes are incorporated into the default 35-member network (See Supplemental material; MINA Workflow In AuD Gene Discovery).

### The AuD network did not arise by chance

IPA calculated the probability that the AuD network could arise by chance by using nine “randomly matched” seed genes. When compared to random permutation, the 35-member AuD network was found to be statistically significant with a score of 26 (p < 10^−26^), where a score of 2 indicates there is a 1/100 chance that the observed network would occur by chance (p < 0.05; 99% confidence level). Gene Ontology comparisons of the 35-network members with all genes in the IPA interaction database suggest that the seed genes within the network showed a higher connectivity than expected by chance. For example, comparison of the network connecting genes revealed that all connecting genes have well-established roles in cell-to-cell signaling (p < 5.28 × 10^−17^) and interactions (p < 1.0 × 10^−15^), cellular development (p < 2.44 × 10^−14^) and immunological diseases (p < 3.19 × 10^−13^). These results suggest highly interacting 26 loci with the nine seed genes beyond what is expected by chance, and the common cellular location and biological function and that common risk variants encoded by members of highly connected networks might possibly impact the function of a few connected genes in the same network and predispose to similar disease etiology or similar disease process.

### GWAS datasets

GWAS databases of diseases of interest (e.g. AuD) were requested and obtained for each disease from its respective source.

### Statistical analysis

The originally “corrected” genetic association p-values were extracted from the GWAS database of interest. We estimated the number of independent SNPs for each gene, using pairwise linkage disequilibrium (LD) between SNPs. HapMap and 1000-genome (http://www.internationalgenome.org/) data were used to estimate LD (D´ of 0.8). We applied a Bonferroni correction, based on the number of independent SNPs we tested, to all previously extracted p-values less than 0.05 and reported the smallest corrected and uncorrected p-value for each gene (extracted from the original study), the total number of valid SNPs genotyped, and the total number of SNPs per gene with p-values less than 0.05. To evaluate evidence of genotype-phenotype association, we selected the smallest adjusted p-value and applied a Bonferroni correction based on the number of independent SNPs we genotyped.

### SNP genotyping

Identified SNPs from GWAS that showed significant association in any database were tested for their association in a second dataset and/ or re-genotyped in new samples.

## References

[1] Kara S., Pirela-Morillo G.A., Gilliam C.T., Wilson G.D. (2019). Identification of novel susceptibility genes associated with seven autoimmune disorders using whole genome molecular interaction networks. J. Autoimmun..

[2] Yin T., Chen S., Wu X., Tian W. (2017). GenePANDA-a novel network-based gene prioritizing tool for complex diseases. Sci. Rep..

[3] Wang X., Gulbahce N., Yu H. (2011). Network-based methods for human disease gene prediction. Brief. Funct. Genom..

[4] Barrenas F., Chavali S., Holme P., Mobini R., Benson M. (2009). Network properties of complex human disease genes identified through genome-wide association studies. PLoS One.

[5] Lettre G., Rioux J.D. (2008). Autoimmune diseases: insights from genome-wide association studies. Hum. Mol. Genet..

[6] Campbell A.W. (2014). Autoimmunity and the gut. Autoimmune Dis..

[7] Podolsky D.K. (2002). Inflammatory bowel disease. N. Engl. J. Med..

[8] Stracke S., Haseneyer G., Veyrieras J.B., Geiger H.H., Sauer S., Graner A. (2009). Association mapping reveals gene action and interactions in the determination of flowering time in barley. TAG Theor. Appl. Genet..

[9] Weersma R.K., Stokkers P.C., van Bodegraven A.A., van Hogezand R.A., Verspaget H.W., de Jong D.J. (2009). Molecular prediction of disease risk and severity in a large Dutch Crohn’s disease cohort. Gut.

[10] Cargill M., Schrodi S.J., Chang M., Garcia V.E., Brandon R., Callis K.P. (2007). A large-scale genetic association study confirms IL12B and leads to the identification of IL23R as psoriasis-risk genes. Am. J. Hum. Genet..

[11] Wellcome Trust Case Control C (2007). Genome-wide association study of 14,000 cases of seven common diseases and 3,000 shared controls. Nature.

[12] Ashtiani M., Salehzadeh-Yazdi A., Razaghi-Moghadam Z., Hennig H., Wolkenhauer O., Mirzaie M. (2018). A systematic survey of centrality measures for protein-protein interaction networks. BMC Syst. Biol..

[13] Winterbach W., Van Mieghem P., Reinders M., Wang H., de Ridder D. (2013). Topology of molecular interaction networks. BMC Syst. Biol..

