# Supplementary material for: Molecular Interaction Network Approach (MINA) identifies association of novel candidate disease genes
Source: MethodsX. 2019 May 31;6:1286–91. doi: 10.1016/j.mex.2019.05.031 (PMC6555892; doi:10.1016/j.mex.2019.05.031)
Supplement: Supplementary file 1 [file mmc1.docx]

**Supplementary material *and/or* Additional information**

One of the major challenges in human genetics today is to understand the genetic architecture of common heritable traits and disorders in humans. With the advent of powerful genome wide association studies (GWAS) and large multi-site collaborative study designs there has been an increase in statistically significant genotype-common phenotype correlations, however, the relative risk of disease imparted by individual genotypes tends to be very small and consequently of limited predictive value. Understanding the relationships between multigene genotypes and disease risk will be essential steps toward the delivery of personalized medicine. We predict that the multigene contributors of many common heritable disorders will map more proximal to one another than predicted by chance within different, whole genome molecular interaction databases, and that consequently network proximity, a measure of the number of molecular interactions separating individual molecules within a molecular interaction network, can be used in combination with GWAS data to explore the genetic architecture of complex human disease. In order to test this hypothesis, we targeted commonly known heritable AuD characterized by relatively high convergence of genotype-phenotype predictions across different GWAS studies.

Over 80 autoimmune disorders have been described, and an alarming rise in the incidence and prevalence is pronounced of more commonly known autoimmune disorders such as Crohn's disease (CD), rheumatoid arthritis (RA), multiple sclerosis (MS), and type I diabetes (T1D)[[6](#_ENREF_6)]. Evidence from independent genetic studies implicates set of genes as candidate genes for mutations that predispose individuals to one or more AuD. One of which is CD, a common idiopathic inflammatory bowel disease (IBD)[[7](#_ENREF_7)] characterized by high heritability (λs ~ 20-35)[[7-9](#_ENREF_7)] and overlapping genetic etiology with other autoimmune-inflammatory conditions including psoriasis[[10](#_ENREF_10)], ankylosing spondylitis[[11](#_ENREF_11)], and T1D [[11](#_ENREF_11)].

Previously published positional cloning or GWAS converge upon the prediction and replications of genes or genetic loci that contribute to the predisposition to AuD. These findings provides an ideal test case to evaluate whether MINA can be used to inform the search for multigenic determinants of common heritable disorders[[12](#_ENREF_12), [13](#_ENREF_13)]. To test our hypothesis, a set of nine AuD “seed genes” were selected based on the results of 18 published GWAS [[5](#_ENREF_5)]. Each of the nine AuD seed genes as associated with two or more of the following seven AuD: Celiac disease (CeD), Psoriasis (PSO), CD, MS, RA, systemic lupus erythematosus (SLE) and T1D [[5](#_ENREF_5)]. We reasoned that this set of nine genes share (or are enriched for) the common biological property that inherited alteration of DNA sequence in or around known coding-DNA segments that predisposes the bearer to each studied AuD. We tested, using three commercially available network-generating software programs and two different whole genome molecular interaction databases Ingenuity Pathway Analysis (IPA), GeneGO Metacore, and Pathway Studio whether this set of 9 genes are located more proximal to one another in whole-genome molecular interaction databases than predicted by chance. The GeneGO Metacore software package was less robust and informative than both IPA and Pathway studio. Furthermore, Pathway studio provided similar data to IPA but with less interaction and interaction evidence than IPA (Data not shown). Secondly, we investigated whether the minimal set of “connecting genes”, required to link the maximal number of seed genes, would be enriched as candidate genes for each individual AuD-predisposing mutations. We provide evidence to support of both these predictions.

**MINA Workflow In AuD Gene Discovery:**

We recently reported the implementation of MINA in identifying association in novel AuD genes [[1](#_ENREF_1)]. The work flow is represented by Figure 1, and the AuD specific network is represented by Figure 2.

1. **Seed genes selection**

The nine seed genes selected included: PTPN22 (CD, T1D, RA, and SLE)*,* IFIH1 (T1D and RA), STAT4 (SLE and RA)*,* IL12A (CeD and T1D), Il2 (CeD, T1D, RA and MS)*,* Il21 (CeD, T1D, RA and MS)*,* IL2RA (T1D and MS)*,* SH2B3 (T1D and CeD) and PTPN2 (T1D and CD).

1. **Ingenuity Pathway Analysis (IPA) core tool created and score-ranked networks interconnecting seed genes**

In MINA, the IPA software was used to examine the connectivity of these nine seed genes among each other which resulted in a single network connecting all nine seed genes. The software was used to build a 35-member network, by default, containing the maximum number of directly connected AuD seed genes. Figure 2 depicts the network from this search that shows a 35-member network comprised of nine seed genes and 26 “network connecting genes”. The Ingenuity database provided information regarding a given gene’s likely cellular location and allows classification of genes and groups of genes by standard gene ontology classifications.

1. **Largest, highest-scoring network from IPA output selected**

The IPA calculated the probability that the AuD network could arise by chance by using nine “randomly matched” seed genes. When compared to random permutation, the 35-member AuD network was found to be statistically significant with a score of 26 (*p*<10^-26^), where a score of 2 indicates there is a 1/100 chance that the observed network would occur by chance (*p<*0.05; 99% confidence level), this indicates that the incorporation of the nine AuD seed genes into a single 35-member network is unlikely to have occurred by chance.

1. **Candidate genes (connecting genes) selection and SNPs identification**

The connecting genes varied depending on the database we used, but over 60% of the network content overlapped between IPA, GeneGo, and Pathway Studio. IPA had few to no “hubs” connecting genes, which made it more robust. The 26 connecting-genes within the developed network and their chromosomal locations are identified using NCBI Gene map ± 100Kbps. We tallied all SNPs within, and immediately surrounding, each of the candidate genes and adjusted the genotype-phenotype correlation scores for multiple testing using a conservative Bonferroni correction.

1. **Validation:** P-values for the 26 connecting gene association are identified from GWAS datasets and the smallest SNP p-value Bonferroni-adjusted by the number of SNPs within the gene. In this study, we interrogated the following GWAS data resources: the Wellcome Trust Case-Control Consortium (WTCCC) and the National Institute of Digestive Disorders and Kidney Disease (NIDDK) (for CD, RA, and T1D); the Celiac Disease study (for CeD); the International Multiple Sclerosis Genomics Consortium (IMSGC) (for MS); the Whole Genome Association Study of Systemic Lupus Erythematosus (SLEGEN) (for SLE). Furthermore, as control, we evaluated the nine seed and 26 candidate connecting genes in non-AuD related samples from the WTCCC data sets, including the WTCCC-type 2 diabetes (WTCCC-T2D) and the WTCCC-Bipolar (WTCCC-BP) data sets. No association was observed for any of the nine seed genes in the WTCCC-T2D and WTCCC-BP data sets. Only one candidate gene (NFKB2) was significantly associated in the WTCCC-T2D data set (rs3802678 p < 7.91 X 10^−6^); and no association with any candidate genes in the WTCCC-BP data set (Data not shown). Furthermore, we evaluated the association of the candidate genes in GWAS of a case-control study of alcoholism, in which the subjects have been drawn from the Collaborative Study on the Genetics of Alcoholism and Parkinson's disease using the resources available at the HuGE and dbGaP. Similar to WTCCC-BP and WTCCC-T2D, only one candidate gene in each GWAS showed association; the v-rel reticuloendotheliosis viral oncogene homolog A (RELA) and the suppressor of cytokine signaling 7 (COS7), respectively. Figure 3 summarizes the data for the presence of the significant SNPs in each disease and list the nine seed genes and 26 connecting network genes (see reference [[1](#_ENREF_1)]; supplemental material), and association results for the previously published and publicly available GWAS.
2. **Replication:** Identified smallest significant SNP allelic association is re-examined in a second GWAS dataset and or in a novel case: control study. To confer risk association replication, we sought to genotype and test the association of the best-associated SNPs in a new AuD samples. We only had sample sizes to test CD and SLE. Although modest, we showed replication of several SNPs within candidate genes to be associated with CD after Bonferroni adjustment [[1](#_ENREF_1)].
